# A Systematic Review of Neurological Manifestations of COVID-19

**DOI:** 10.7759/cureus.28309

**Published:** 2022-08-23

**Authors:** Sumeru Thapa Magar, Hatim I Lokhandwala, Saima Batool, Faiqa Zahoor, Syeda Kisa Fatima Zaidi, Saveeta Sahtiya, Deepa Khemani, Sumeet Kumar, Diana Voloshyna, Faraz Saleem, Muhammad Abu Zar Ghaffari

**Affiliations:** 1 Cardiovascular, Janaki Medical College & Teaching Hospital, Tribhuvan University, Kathmandu, NPL; 2 Internal Medicine, Jinnah Postgraduate Medical Centre, Karachi, PAK; 3 Internal Medicine, Hameed Latif Hospital, Lahore, PAK; 4 Medicine and Surgery, Al-Ameen Medical College, Bijapur, IND; 5 Medicine and Surgery, Jinnah Sindh Medical University, Karachi, PAK; 6 Pediatric, Chandka Medical College, Kashmore, PAK; 7 Medicine, University of Michigan, Ann Arbor, USA; 8 Internal Medicine, Akhtar Saeed Medical & Dental College, Lahore, PAK

**Keywords:** covid-19, central nervous system (cns), peripheral nervous system (pns), sars-cov-2, neurological manifestations

## Abstract

The coronavirus can infect the upper respiratory tract, sinuses, and nose, and its severity manifests in its respiratory symptoms and neurological and psychological consequences. The majority of people who have COVID-19 present with moderate flu-like illness, and patients who are elderly with comorbid conditions, such as hypertension and diabetes, are more prone to experience severe illness and death. However, in the ongoing COVID-19 pandemic, neurological consequences have become a substantial source of morbidity and mortality. COVID-19 poses a global hazard to the nervous system because of its widespread dispersion and multiple pathogenic pathways. This review offers a critical assessment of the acute and long-term neurological effects of the COVID-19 virus. Some neurological problems include headache, dizziness, myalgia/fatigue, meningitis, ischemic/hemorrhagic stroke, and myelitis. Other people who have contracted COVID-19 also exhibit neurological features such as loss of taste and smell, reduced consciousness, and Guillain-Barré syndrome. This study seeks to help neurologists comprehend the wide range of neurologic aspects of COVID-19, as understanding neurological symptoms may help with the management and enhance the patient's outcomes.

## Introduction and background

The city of Wuhan in China initially identified COVID-19 in a patient on December 8, 2019, as atypical pneumonia. Research has revealed that severe acute respiratory syndrome coronavirus type 2 (SARS-CoV-2) causes this coronavirus disease [1]. SARS-CoV-2 is a unique novel coronavirus with a more extended incubation time, a shorter serial gap, and a lower fatality rate, making it more contagious [2]. Initially, it began as an outbreak, and the World Health Organization (WHO) proclaimed it a Public Health Emergency of International Concern on January 30, 2020 [3]. The primary concern behind this coronavirus was its rate of transmission and the number of deaths that culminated. COVID-19 became an epidemic, with 44,672 cases confirmed in China by February 14, 2020 [3,4]. It quickly expanded outside of China to the world, primarily through airborne droplet transmission from person to person. The World Health Organization (WHO) declared COVID-19 a pandemic on March 11, 2020 [3], due to the high rate of the subsequent increase in cases. At this point, it had infected more than a million people worldwide. According to estimates, during the epidemic's early stages, the number of infected people doubled every two days, and 4.7% to 6.6% of people [5] might contract the disease from a single infected person. As of September 9, 2020, there had been over 19,851,252 deaths globally from the COVID-19 infection, which has spread to 216 countries [3]. According to early data from China, 81% of patients experienced minor symptoms only. In comparison, 5% of patients reported experiencing severe illness, such as respiratory failure and shock. According to reports, the disease's overall fatality rate is 2.3% and higher in the elderly [1,3,6].

In most cases, the infection causes a dry cough, fever, dyspnea, sore throat, and severe respiratory involvement in elderly individuals. While most of those infected exhibit mild or no symptoms, some people develop pneumonia, meningitis, and multiple organ failure. More than 35% of COVID-19 patients reportedly experience neurological problems [7], and neurological symptoms may be the first signs of the disease in specific COVID-19 individuals. However, patients with severe COVID-19 infections are more likely to experience neurological signs and symptoms, which may be the result of cerebral hypoxia brought on by respiratory failure [8].

This review investigates COVID-19's neurological manifestations and potential risk variables linked to the emergence of neurological symptoms and signs. It divides the neurological symptoms and signs of those linked to the central nervous system (CNS) and those linked to the peripheral nervous system (PNS). Understanding neurological symptoms may help physicians with early diagnosis and management of COVID-19 and improve the patient's outcomes.

## Review

Methods

**Figure 1:**
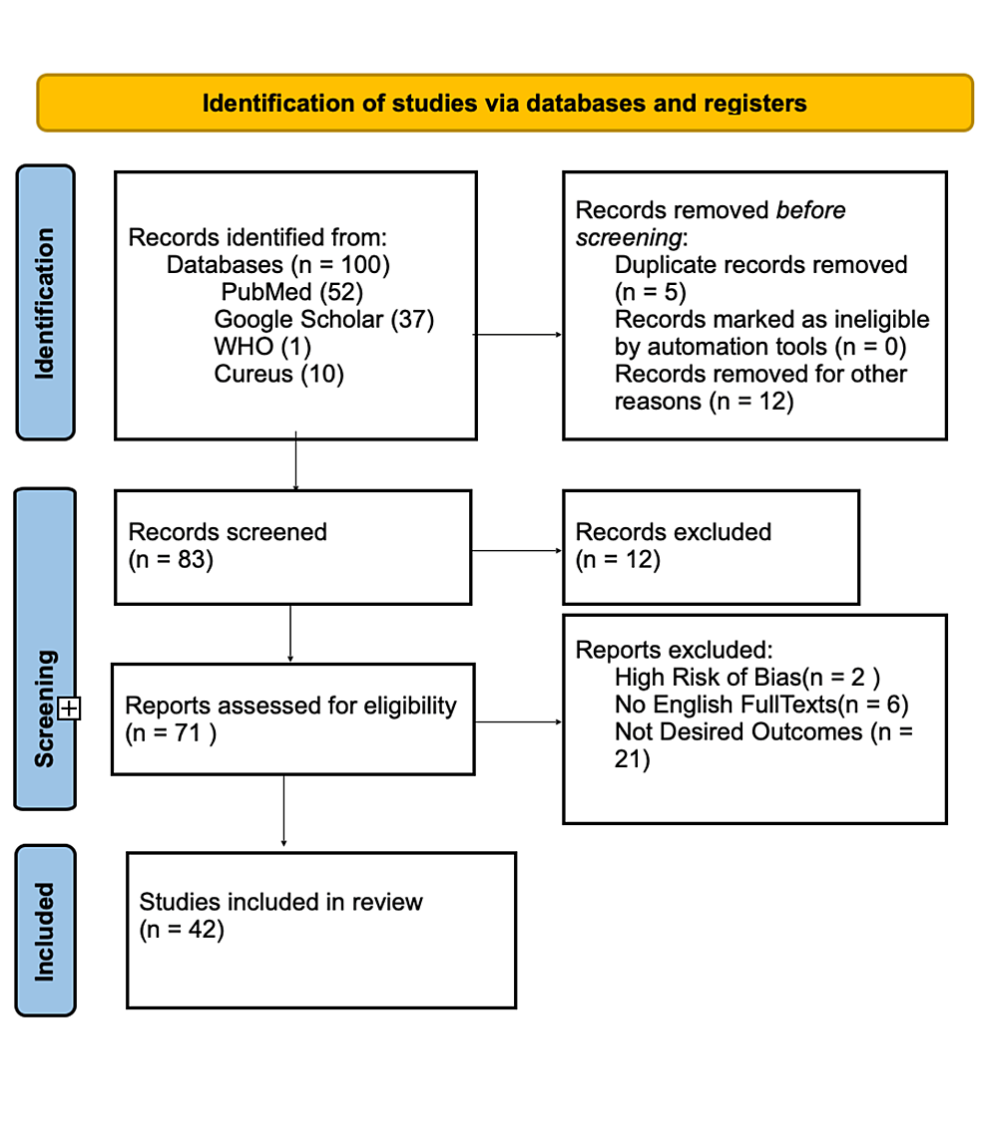
Preferred Reporting Items for Systematic Reviews and Meta-Analyses (PRISMA) Flow Chart

The study used the following methodological framework in conjunction with the extended Preferred Reporting Items for Systematic Reviews and Meta-Analyses (PRISMA) checklist for scoping reviews (Figure 1).

Search Strategy and Selection Criteria

The systematic review includes observational retrospective case studies and research articles about individuals with proven COVID-19 infection and its neurologic symptoms from Google Scholar, PubMed, Cureus, and the World Health Organization database to establish an extensive pool of helpful information regarding the neurological effects of COVID-19. It includes some gray material and literature for the same purpose. We deliberately placed a search limit after December 2019, as that is when the first COVID-19 case surfaced. Some straightforward terms used for the search were "Coronavirus," "COVID-19," and "SARS-CoV-2" alone or in combination with "neurology," "neuroinvasion," "CNS," "PNS," "meningoencephalitis," "stroke," "Guillain-Barre syndrome (GBS)," "neuromuscular disorder," and "Cerebrovascular Disease-Related Symptoms."

Inclusion and Exclusion Criteria

We used cross-referencing to find any missing pertinent articles and identified neurological symptoms of COVID-19 and its underlying mechanisms as the main themes. The study included a population of all ages and sexes and articles from various parts of the world. If the papers dealt with COVID-19-positive humans, we had the full texts in English. The study omitted thematically unrelated research, animal studies, editorials, commentaries, case reports, and preprints. Instead, it focused on peer-reviewed case-control, population-based cohort studies or human clinical trials.

Data Extraction and Quality Assessment

We checked all titles and abstracts and included entire texts after discussion and 100% agreement over the papers. Variables that aided in data extraction were information about the research (name of author(s), year of publication, title), methodology (study design or type), and intervention (duration and setting). The Joanna Briggs Institute (JBI) tool was also utilized, as it offers thorough criteria for the assessment and evaluation of the majority of study types and offers thorough checklists for the evaluation and assessment of the majority of study types. Therefore, by using JBI’s checklist methodology for cohort studies, case series, and case reports, we can solidify the analysis and understanding of this systematic review’s findings.

Results

In total, 100 articles were reviewed, and after quality assessment, 58 were excluded. Forty-two articles were included in this review: retrospective case series, case reports, prospective case series, multicohort and multicenter studies, and systematic review articles about individuals with proven COVID-19 infection and its neurological symptoms. The sample sizes ranged from a single patient to 3,055 patients [9-12]. Several neurological signs were found, with the most prevalent neurological complaints overall being fatigue (32%), myalgia (20%), taste impairment (21%), headache (19%), and impairment of smell (21%) [13]. The study also found that stroke is the most common diagnosis of COVID-19, and we included five other sample studies to support this [2,8,14-16]. Two other studies supported dizziness as a neurological symptom [13,16], while six other articles supported headaches as a common symptom. We did not base our study in one area, and the articles were from all over the world. We even included studies from China [17] and the US [18]. Two of the articles were specific studies, with one focusing on a 24-year-old [11], while the other focused on a 41-year-old [12]. Hence, it is expected that their results mirror those of other case reports and study series. One of the articles summarized and pooled information on neurological signs and symptoms from 350 studies [13] (Table 1).

**Table 1:** Pooled Prevalence of Neurological Symptoms The pooled prevalence of neurological symptoms (Misra et al. [13]).

Variables	Number of Studies	Pooled Events	Pooled Sample Size	Pooled Prevalence
Agitation	3	145	468	45
Fatigue	169	14,121	45,766	32
Myalgia or fatigue	22	619	2,246	31
Taste Impairment	38	2,934	12,631	21
Myalgia	207	12,183	59,821	20
Smell impairment	51	4,647	30,925	19
Smell/taste impairment	14	518	3,100	18
Headache	202	8,609	51,969	13
Headache/dizziness	9	676	3,520	12
Seizure	15	127	15,467	1
Disturbance of consciousness	25	693	15,129	7
Dizziness	46	809	13,473	7

Characteristics of the Results

Table 2 outlines a summary of the case series reviewed according to the type, sample size, and findings of the eight studies [10,15-21]. This evidence demonstrates that SARS-CoV-2 may affect the body’s central nervous system.

**Table 2:** Summary of the Studies Reviewed SARS-CoV-2: severe acute respiratory syndrome coronavirus type 2.

No.	Authors	Study Type	Sample Size	Focus of Study	Study Findings
[10]	Chou et al. (2021)	Multicohort study	3,055	To identify the neurological characteristics, prevalence, and consequences in individuals admitted with COVID-19.	Persons admitted with SARS-CoV-2 frequently had neurological symptoms, and these symptoms were linked to greater in-hospital mortality. In SARS-CoV-2, neurological disorders already existent were linked to a higher chance of developing neurological symptoms and syndromes.
[17]	Huang et al. (2020)	Prospective case series	41	To determine the clinical characteristics of COVID-19 patients in Wuhan, China.	The infection resulted in clusters of severe respiratory illnesses resembling severe acute respiratory syndrome coronavirus which was linked to ICU hospitalization and high mortality.
[19]	Xu et al. (2020)	Retrospective study series	62	To research the clinical traits of individuals with the 2019 SARS-CoV-2 disease in Zhejiang Province, China.	The manifestations of the patients in Zhejiang province are very moderate when compared to those of the COVID-19 patients who were initially afflicted in Wuhan.
[15]	Pilotto et al. (2021)	Multicenter study	41	To illustrate the clinical phenotypic, lab, and neuroimaging discoveries of encephalitis caused by the SARS-CoV-2 disease, their connection to respiratory function and inflammatory markers, and their progression in terms of clinical manifestations and response to treatments.	Findings highlighted that encephalitis has a broad clinical spectrum linked to COVID-19 and underlies several pathophysiological processes. Specific central nervous system (CNS) manifestations had a significant impact on the patient's treatment response and end outcome.
[18]	Benameur et al. (2020)	Prospective case series	3	To study encephalopathy and encephalitis linked to COVID-19 illness and changes in cerebrospinal fluid cytokine levels.	Patients with neurologic sequelae can show SARS-CoV-2 antibodies in their CSF, and these antibodies are linked to specific CSF cytokine changes.
[20]	Klopfenstein et al. (2020)	Retrospective observational case series	54	To outline anosmia's prevalence and characteristics in coronavirus patients.	Fifty percent of the study's European coronavirus patients had anosmia, which was frequently accompanied by dysgeusia.
[21]	Lechien et al. (2020)	Multicenter study	417	To research the presence of gustatory and olfactory dysfunctions in individuals with coronavirus disease that has been verified in the laboratory.	European patients with the coronavirus are more likely to experience gustatory and olfactory abnormalities than nasal symptoms.
[16]	Mao et al. (2020)	Retrospective observational case series	214	To investigate the neurologic symptoms that coronavirus patients experience.	Commonly, COVID-19 patients exhibit neurologic symptoms.

Discussion

The sharp rise in COVID-19 verified cases and deaths is evidence of person-to-person transmission by the virus. According to the Centers for Disease Control and Prevention, the virus transmits by person-to-person contact (within six feet) via respiratory droplets [9]. Due to the neurotropic properties of the virus, COVID-19 affects the central and peripheral nervous systems (CNS and PNS), either directly or indirectly. These include thrombosis complications, inflammatory side effects, hypoxia, metabolic anomalies, and labile blood pressure. Neurological signs and symptoms involving the central or peripheral nervous systems are either self-reported symptoms (such as headache and ageusia) or neurological signs or diagnoses obtained through clinical evaluation [10]. Generally, viral pathogens can enter the central nervous system (CNS) through various routes, including the hematogenous route, which involves endothelial infection, and peripheral nerve or olfactory neuron paths [22,23]. Epithelial cells in the digestive and respiratory tracts are the primary target cells for SARS-CoV-2 [24].

The SARS-CoV-2 virus may harm these cells by attaching them to angiotensin-converting enzyme-2 (ACE-2), reducing mitochondrial function and endothelial nitric oxide synthetase activity, which can indirectly affect the heart and brain. The angiotensin-converting enzyme employs angiotensin-converting enzyme-2 (ACE-2) receptors in these cells to allow a virus to enter the cells. SARS-CoV-2 can invade and harm angiotensin-converting enzyme-2 (ACE-2) receptors, leading to neurological consequences. One neurological manifestation is harm to the brain through a cytokine storm brought on by the immune system's response to the virus. It moves throughout the body and crosses the blood-brain barrier, causing infections in the brain and surrounding neurons and glial cells [25].

The blood-brain barrier and the blood-cerebrospinal fluid barrier normally protect the brain from the unrestricted passage of unwanted chemicals, viruses, and cells [24]. Brain damage may also result from cytokine release due to microglial activation and a generalized inflammatory response [14]. For example, proinflammatory cytokines with high levels in the bloodstream cause altered mental status [26,27]. Since the angiotensin-converting enzyme-2 (ACE-2) receptor controls the renin-angiotensin-aldosterone system (RAS), disrupting it can cause labile blood pressure [28].

The neurological consequences also manifest through hypoxia and lack of oxygen reaching the brain. Ischemic strokes can also result from thrombosis problems. Given that SARS-CoV-2 may affect the central nervous system (CNS) directly or indirectly, acute cerebrovascular illness, stroke, and intracranial infection-related symptoms are among the CNS-related manifestations linked to COVID-19 that this review explains. In 13 cases of unexplained encephalopathy, MRI brain scans showed bilateral frontotemporal hypoperfusion in 84% of the cases, leptomeningeal enhancement in 62% of the cases, and ischemic stroke in 23% of the cases [16,29].

Nonspecific neurological signs and symptoms

Patients with COVID-19 most frequently reported nonspecific symptoms, including headaches, fatigue, dizziness, and nausea.

Dizziness

In 46 case studies, dizziness had a prevalence of 7% (95% CI: 5% to 8%), and in nine other studies, headache and dizziness occurred together with a prevalence of 12% (95% CI: 8% to 17%) [13]. It is a generic symptom of many illnesses and is one of the most prevalent neurological characteristics of COVID-19, especially in patients receiving intensive care due to serious illness [16].

Headache

The most prevalent CNS symptom is headache, with prevalence rates ranging from 6.5% to 23% and a mean of 8% in several studies [7]. In a Wuhan study, 8% of patients reported headaches as a symptom [17], whereas a study from Zhejiang found that 34% of patients had headaches [19]. In another French study, 82% of the COVID-19 cases had a headache as a symptom [12]. A recent study of 130 hospitalized COVID-19 patients found that 35% had severe headaches with a frontal predominance and an oppressive nature. The study revealed that 62% of those patients suffered headaches within 24 hours of contracting the disease. Nearly half of the individuals exhibited tension-type headaches [11]. Typically, migraines, tension-type headaches, and acute headaches brought by flu-like sickness predominate in the first few days of illness. Headaches brought on by hypoxia and systemic inflammation due to a cytokine storm may develop later. In venous sinus thrombosis and meningitis associated with COVID-19, headaches may potentially be sentinel signs [2].

CNS-specific neurological signs and symptoms

Cerebrovascular Disease-Related Symptoms

There have been numerous reports of COVID-19-related cerebrovascular instances, such as acute ischemic stroke. A case study by Mao et al. (2020) on 214 COVID-19 patients reported six (2.8%) stroke cases (five ischemic and one hemorrhagic) [16]. A different group of 221 hospitalized COVID-19 patients revealed 13 (5.8%) stroke cases (11 (5%) ischemic and one (0.5%) instance of hemorrhagic stroke) [16]. Extensive research by Misra et al. (2021) also revealed that stroke was present in 29 studies [13]. The pooled prevalence was 2% (95% CI: 1% to 2%), with hemorrhagic stroke at 0.31% (95% CI: 0.15% to 0.50%, 21 studies), ischemic stroke at 1% (95% CI: 1% to 2%), and cerebral venous thrombosis at 0.12% [13]. Typically, ischemic stroke manifests after 12 days of COVID-19. Significant vascular obstruction is a common cause of COVID-19-related ischemic stroke [2]. Older patients and patients in critical condition or those in the ICU will have the typical risk factors for cerebrovascular illness. Decreased platelet counts suggest that hypertensive patients may have elevated blood pressure and a higher risk of cerebrovascular hemorrhage. There is also a strong tendency for clot formation in some COVID-19-positive critically ill individuals [15], and some have experienced cerebral venous thrombosis (CVT) [14].

Altered Mentation

Approximately 9% of people who have contracted COVID-19, especially those who are severely ill, may have impairments in their degree of consciousness [30]. Older people, particularly those with preexisting chronic medical conditions, are more likely to experience delirium or impaired consciousness. Almost one-third of patients may experience dysfunction after discharge, and more than two-thirds of critically ill patients exhibit agitation and disorientation [18]. These patients may present with encephalopathy and confusion. Furthermore, cerebral hemorrhages may cause altered mental status. Toxic-metabolic encephalopathy brought by systemic hyperinflammation, cerebrovascular events, seizures, and a potential SARS-CoV-2 CNS infection causes altered mentation in COVID-19 [29,30].

Seizures

Patients with COVID-19 have experienced recurrent transient generalized seizures and epilepsy. Most instances, if not all, had a history of seizure disorders or epileptic seizures. In a group of 32 COVID-19 patients who experienced seizures, 40% had no prior diagnosis of epilepsy or other disorders affecting the central nervous system [14].

A systematic examination of case series and reports found that 47 patients with COVID-19 experienced epilepsy. Most individuals did not have a history of previous seizures and had previous respiratory problems. COVID-19 severely affects the central nervous system because of a cytokine storm brought on by proinflammatory cytokines entering the CNS from the periphery or produced by activated microglia. Tumor necrotizing factor, granulocyte colony-stimulating factor, and the release of inflammatory cytokines are among the hypotheses to explain COVID-19-associated epilepsy. These factors can cause neuronal hyperexcitability by activating glutamate receptors, resulting in episodic seizures. Secondary seizures may develop in COVID-19 patients from strokes, electrolyte abnormalities, and elevated oxidative stress [30]. On the other hand, some scientists think that encephalitis and virus invasion of the brain may be to blame for seizures. In COVID-19 patients, a lower seizure threshold may develop even without overt inflammatory signs, leading to new-onset seizures or the recurrence of previously well-controlled seizures. However, in some circumstances, an adverse pharmacological reaction from antiviral medications such as ribavirin may cause epilepsy [14].

Meningoencephalitis

Thirty-three cases of meningoencephalitis from SARS-CoV-2 infection report numerous manifestations, including altered sensorium (71%), aphasia/dysarthria (53%), headache (34%), seizures/status epilepticus (34%), focal neurological deficits (18%), and myoclonus (9%) [2]. An MRI revealed a right mesial temporal lobe signal abnormality in a 24-year-old patient who experienced seizures, impaired mental state, headaches, exhaustion, and fever for a few days. Twelve mononuclear cells and two polymorphonuclear cells were also reported in the cerebrospinal fluid (CSF) of the patient with SARS-CoV-2 [31]. CSF analysis showed pleocytosis and positive viral polymerase chain reaction (PCR) in a 41-year-old patient who experienced a seizure and altered mental status [12,17].

PNS-associated signs and symptoms

Anosmia and Ageusia

Scent problems and taste disorders are common in some people who have contracted the coronavirus. Over 80% of patients without nasal blockage or discharge report acute intermittent impairment in odor. The precise mechanisms underlying SARS-CoV-2 anosmia and ageusia are still unclear. Although olfactory dysfunction manifests concurrently with or after the clinical beginning of COVID-19 in 88% of cases, it could be the sentinel sign in 12% of cases [2].

The majority of the time, anosmia and ageusia are present in asymptomatic people or when the disease first manifests itself without any other symptoms. As individuals recover from SARS-COV-2 infection, most patients progressively regain their sense of taste and smell. Although other people's recovery from smell and taste impairment may take longer, 25% to 80% of instances have shown near complete resolution in under two weeks [20,32].

Guillain-Barré Syndrome

After COVID-19 infection, people around the world documented a few isolated cases of Guillain-Barré syndrome (GBS). At least 73 instances of GBS and its variations associated with COVID-19 have been documented thus far [21]. The most common clinical manifestation (70%) was flaccid-areflexic weakness with or without sensory complaints, while 10% of cases had characteristics indicative of Miller-Fisher syndrome (MFS). There have also been reports of polyneuritis cranialis (2.7%), facial diplegia (6.8%), and pharyngeal-cervical-brachial variation (1.3%) [33]. There are two reports of isolated right abducens-related ophthalmoplegia and pupillary-sparing oculomotor nerve palsy [34]. Interestingly, one-fourth of the patients had early and severe respiratory impairment, probably caused by the brainstem respiratory center's involvement [35]. Most of these individuals showed progressing limb weakness that worsened over one to four days [36]. Similar to other viral infections linked to GBS, there is a five- to 16-day lag between the onset of viral illness and the emergence of muscle weakness [33].

While neurological symptoms typically started to arise during the first week of illness and were indicative of an immune-mediated postinfectious pathology, one case had a neurological beginning indicative of autoimmune para-infectious pathology. However, it is unclear whether COVID-19 is more likely to cause GBS. Although numerous studies have discovered a connection between SARS-CoV-2 infection and GBS, further information is required to validate the association and pinpoint the precise mechanism. Researchers should assess epidemiological data about the alleged infectious agent and GBS to validate the link between COVID-19 and GBS. Direct viral invasion may not be as important in developing COVID-19-related GBS as indirect immune-mediated mechanisms such as neuroinflammation and molecular mimicry. Additionally, additional molecular analysis is required to pinpoint the precise process that results in GBS following SARS-CoV-2 infection [37].

Muscle Involvement

Since angiotensin-converting enzyme-2 (ACE-2) is present in skeletal muscle, SARS-CoV-2 may associate with it and infect the muscle. SARS-CoV-2 can directly target the nervous system by binding to angiotensin-converting enzyme-2 (ACE-2) and inflicting skeletal muscle injury, or it can enter the central nervous system via peripheral nerves. Skeletal muscle damage is a sign of nervous system dysfunction. Myalgia may result from injury to the skeletal muscles. Myalgia shows muscular pain and discomfort from local or systemic infection. Patients with COVID-19 who complained of muscle problems had higher levels of creatine kinase (CK) and lactate dehydrogenase (LDH). This prevalent symptom of COVID-19 manifests in 10%-74% of cases [2].

In one study, 11% of hospitalized COVID-19 cases had skeletal muscle injury with elevated blood creatinine kinase levels > 200 U/L, particularly in patients with hepatic and renal failure [38]. COVID-19 cases document that rhabdomyolysis causes weakness, discomfort, and tenderness in the lower limbs. Some patients experienced weariness, discomfort in their muscles, and higher levels of muscle enzymes, which may indicate virus-induced muscle damage and inflammation [39-41].

## Conclusions

This is the first time in a long time that a virus (the coronavirus) has spread over the globe, and it has caused a significant change in how people do things. The health industry was under a lot of strain trying to limit its spread. Several worldwide health organizations collaborated to battle this disaster. For those in the medical field, the COVID-19 epidemic poses some difficulties that present in various neurological ways, and in many cases, neurological symptoms may appear before normal respiratory symptoms. This review proves that numerous neurological symptoms of COVID-19 are possible. It is essential to have a comprehensive understanding of the range of COVID-19's neurological effects if we want to stop the virus's spread. As the outbreak gradually subsides, the number of postinfectious neurological problems, including GBS, will also decrease. Although neurological symptoms are frequently present in severe cases, neurologists and other medical personnel should diagnose patients with a solely neurological presentation with extreme caution.

Generally, clinicians need to be mindful of potential neurological and cognitive issues following COVID-19, particularly in elderly patients, patients with cognitive impairment, or patients with psychiatric comorbidities. When treating and managing patients with neurological comorbidities, especially those who are using immunosuppressants, medical personnel must take the appropriate caution. The overview of COVID-19's neurological manifestations provided above will aid the neurologist in making the necessary preparations, which are crucial for avoiding infections. People should have a neuropsychological evaluation or cognitive screening if there are cognitive concerns following COVID-19 and consult a psychiatrist or psychologist in cases of complex cognitive or emotional issues following COVID-19.
